# Magnetic field-induced rubber-like behavior in Ni-Mn-Ga particles/polymer composite

**DOI:** 10.1038/s41598-019-40189-2

**Published:** 2019-03-05

**Authors:** P. Sratong-on, V. A. Chernenko, J. Feuchtwanger, H. Hosoda

**Affiliations:** 1 Institute of Innovative Research (IIR), Tokyo Institute of Technology, 226-8503 Yokohama, Japan; 2 Ikerbasque, Basque Foundation for Science, 48013 Bilbao, Spain; 3 BCMaterials, Basque Center for Materials, Applications and Nanostructures, UPV/EHU Science Park, 48940 Leioa, Spain; 4 University of Basque Country (UPV/EHU), 48080 Bilbao, Spain

## Abstract

Single crystalline Ni-Mn-Ga is well known as a prototype ferromagnetic shape memory alloy (FSMA) exhibiting a giant magnetic field-induced strain (MFIS), up to 12%, due to the magnetically driven twin boundary rearrangement. The large stroke and fast magnetomechanical response make it important for actuators and sensors. Polycrystalline Ni-Mn-Ga is inexpensive and technologically easy accessible, but constrains from the grain boundaries inhibit the twin boundary motion, whereby a very low MFIS is observed. Here, we have shown for the first time that a polycrystalline Ni-Mn-Ga can be split into the magnetostrain-active single grains which, being specially assembled in a silicone polymer matrix, caused large and fully reversible MFIS of the resulting composite. We termed the unique reversibility of a large MFIS of the composite as the magnetic field-induced rubber-like behavior. The magnetostrain of individual particles was explored by the X-ray μCT 3D imaging. The results suggest novel solutions for development of the low cost magnetic actuators and sensors for haptic applications.

## Introduction

Bulk Heusler-type Ni-Mn-Ga alloys are well known as the prototype FSMAs that exhibit a giant magnetic field-induced strain (MFIS)^1^. The giant MFIS, equal to 6% and 10% for 5-layered modulated tetragonal and 7-layered modulated orthogonal martensites^2,3^, respectively, as well as 12% for non-modulated tetragonal martensite^4^, can be displayed by the bulk single crystalline Ni-Mn-Ga FSMAs when subjected to a uniform magnetic field below 1T. Such large values of MFIS are the result of a field-driven reorientation of one twin variant into another. A reset to the former twin variant should be done by the orthogonally applied mechanical stress or magnetic field. To date, the most technologically elaborated and studied single crystalline Ni-Mn-Ga alloys are those which exhibit a 5-layered tetragonal martensitic structure. They demonstrate fast variant-switching times (down to the microseconds regime), high resolution in the positioning control and lifelong fatigue making them very attractive for the applications as actuators, sensors, energy harvesters and damping devices^5–8^, although high cost and enhanced brittleness are still the key issues.

Polycrystalline Ni-Mn-Ga alloys could be much more cost efficient solutions for actuator materials than the single crystalline analogs, but very low MFIS, of about 0.1%, and high brittleness impede their application. The principal problem of low MFIS stems from the grain boundaries constraining twin boundary motion inside the grains^9,10^. Partial removal of these constrains and, as a consequence, large MFIS of 8.7%, was achieved in the Ni-Mn-Ga foam, where about half amount of the grains were replaced by the pores^11^. Although Ni-Mn-Ga foam could exhibit large single MFIS, the latter was much reduced, down to about 2%, in a cyclic regime^11^. Alongside a cyclic stability issue, one should take into account that the foam technology of Ni-Mn-Ga is a complicate and time-consuming process.

In the present work, the aforementioned constraints were completely eliminated by disintegration of the polycrystalline Ni-Mn-Ga into the individual grains with a special technique described below. Then, the powder consisting of these grains was used to prepare a magnetostrain-active Ni-Mn-Ga/polymer composite. The well-controlled fabrication process of such a composite is an advantageous approach to handle with the difficulties inherent to the manufacturing methods in the case of FSMA bulk single crystals or foams.

From preliminary experiments and finite elements calculations, one can deduce the prerequisites that are needed to expect a large MFIS from the Ni-Mn-Ga/polymer composite: (i) individual particles should exhibit MFIS, which means that their twinning stress should be lower than the magnetostress (usually about 1 MPa at a saturating magnetic field of less than 1 T)^12^; (ii) the stiffness-matched polymer should be used in order to facilitate particles to deform easily inside a composite under magnetic field; (iii) due to a dilution effect on the MFIS characteristics of composite, one should take care about a large enough volume fraction of particles being compromised with the upper bound limit, where the deformation of particles is blocked by the neighboring particles; and (iv) finally, the spatially-controlled assembling of particles and their crystallographic orientation are needed. An analysis of the available literature shows that the non-fulfillment of the one from the aforementioned conditions resulted in a Ni-Mn-Ga/polymer composite with a negligible MFIS, less than 0.01%^13–15^. On the other hand, the conditions (i)-(iii) are not important when the stress-induced actuation of particles in composite is considered. In this case, the Ni-Mn-Ga/polymer composites serve as the advanced materials for vibration damping, where the stress-induced twin boundary motion is the main mechanism for a large absorption of the mechanical energy^16–18^.

In order to prepare magnetostrain-active Ni-Mn-Ga particles (condition (i)) with a reduced amount of the lattice defects and low surface roughness, which act as the obstacles to twin boundaries motion^19^, we have recently suggested an innovative method of a drastic intergranular embrittlement enabling easy disintegration of the polycrystalline Ni-Mn-Ga alloy into the individual grains^20,21^. The embrittlement was achieved by adding of 0.03 at.% of Bi which entirely segregates to the grain interfaces facilitating an easy cleaving of the Ni-Mn-Ga ingot into single crystalline grains above melting point of Bi^20^. In the present work, this technology was used to synthesize a Ni-Mn-Ga powder. To fabricate composite, the powder with a 30 vol.% fraction was embedded into a silicone polymer (ELASTOSIL M4400) with the elastic stiffness below 1 MPa, which is comparable to the twinning stress of the particles. So, the conditions (ii) and (iii) were satisfied. Then, the composite was cured under magnetic field to let particles align in the columns with their crystallographic easy magnetization axis oriented along the field direction (condition (iv)). Thus, all the conditions for large MFIS in this composite have been met in the present work which led to the outstanding nontrivial results described below.

## Results

### Martensitic transformation and microstructure of Ni-Mn-Ga particles. Magnetization behavior of Ni-Mn-Ga/silicone composite

**Figure 1:**
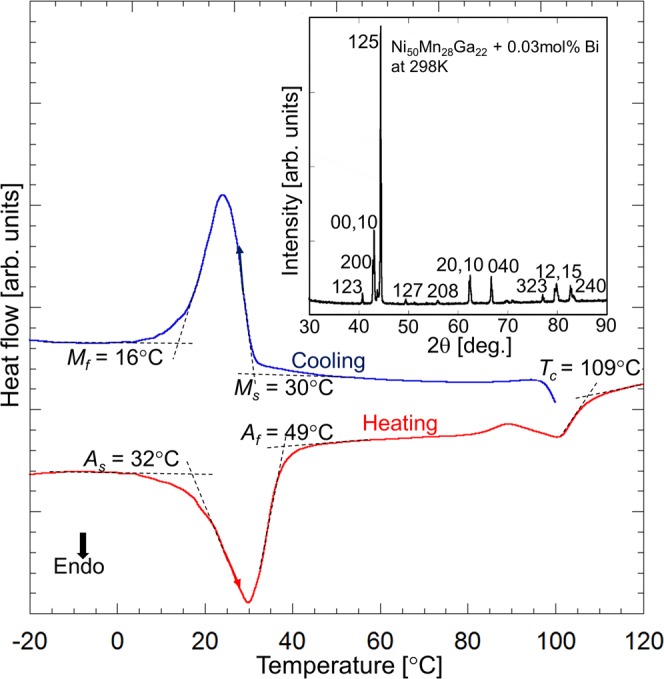
Calorimetric dependences of Ni-Mn-Ga powder during cooling-heating. Characteristic temperatures of martensitic transformation and Curie temperature are indicated. The inset presents X-ray powder diffraction pattern at room temperature, CuK_α_.

The magnetostrain-active Ni-Mn-Ga particles, representing individual single crystalline grains, were prepared by the aforementioned innovative procedure (see also Methods for details). Figure 1 shows the calorimetric heating-cooling dependences for Ni-Mn-Ga powder measured by the differential scanning calorimetry. The transformation temperatures, equal to 30 °C and 16 °C for martensite start and finish temperatures, 32 °C and 49 °C for austenite start and finish temperatures and 109 °C for the Curie temperature, were determined from the calorimetric curves by a standard two-tangents method, as shown in Fig. 1. The inset to Fig. 1 presents the X-ray diffraction pattern of Ni-Mn-Ga powder. The spectrum fits well to the 5-layered martensitic structure with the lattice parameters of the pseudo-tetragonal unit cell *a* = *b* = 5.94 Å, *c* = 5.60 Å and c/a = 0.94, in agreement with the literature results^22,23^.

**Figure 2:**
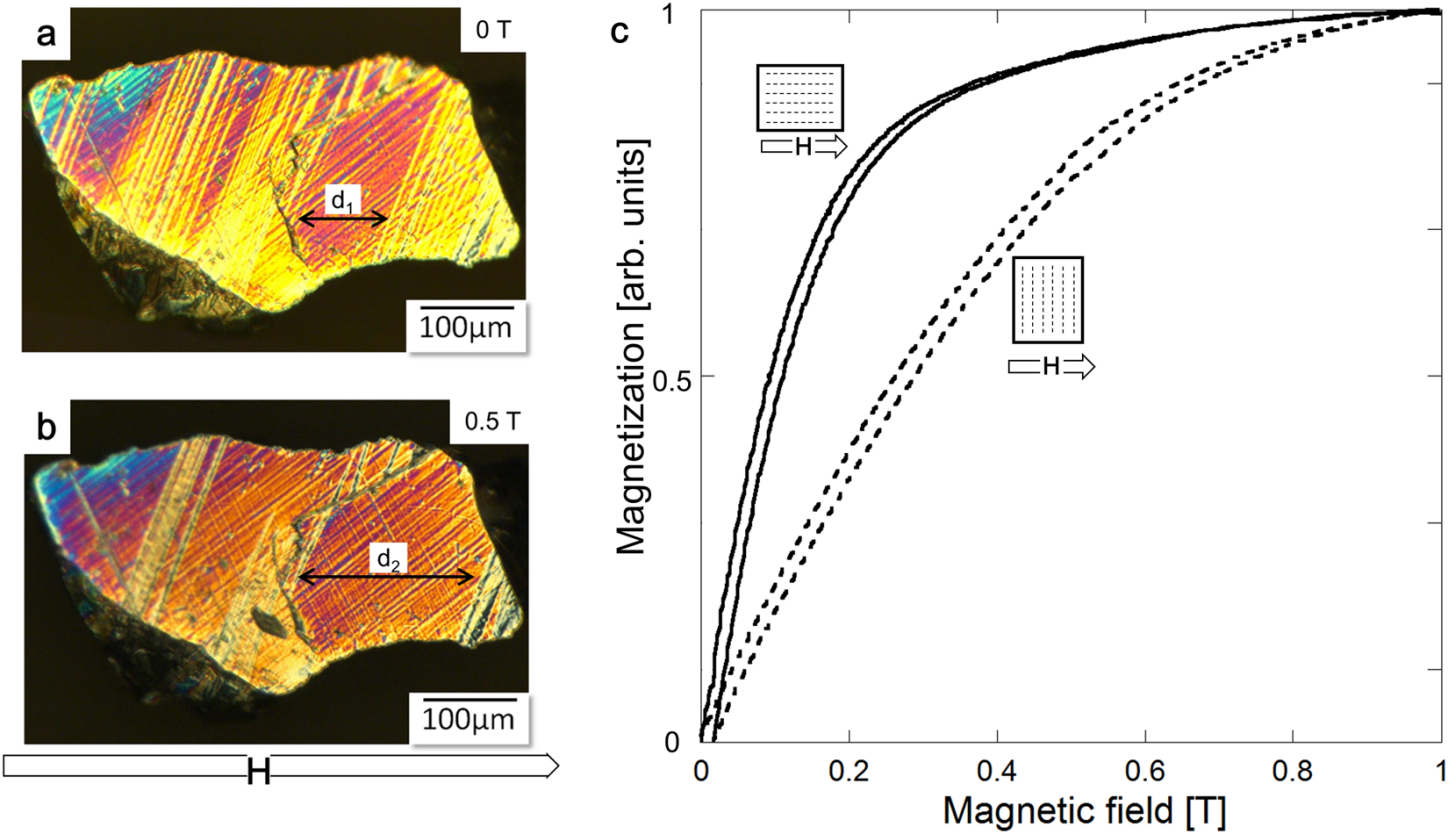
Optical micrograph of the polished surface of Ni-Mn-Ga single crystalline particle showing internally twinned martensitic variants: (**a**) without magnetic field and (**b**) under magnetic field, μ_0_H = 0.5 T. Magnetic field induced twin boundary motion increases the thickness of the favorably oriented variants, d_2_ > d_1_. (**c**) Magnetization curves of Ni-Mn-Ga/silicone composite normalized to the magnetization value at 1 T. The curves demonstrate easy and hard magnetization directions of composite located along and perpendicular to particles chains, respectively, indicating a preferable crystallographic orientation of the Ni-Mn-Ga tetragonal unit cell with short *c*-axis along the particles chains.

Figure 2a presents an optical image of the cross-sectional view of one-grain-particle showing a typical martensitic structure, where twin boundaries extend across a single crystal. Figure 2b shows that the volume fraction of twin variants with the easy-magnetization *c*-axis along the applied magnetic field of 0.5 T considerably increases on the expense of variants with *c*-axis perpendicular to the direction of magnetic field, which proves that the particle is magnetostrain-active. The total thickness increase of favorably oriented twin variants, due to a magnetic field induced twin boundary motion, was estimated from Fig. 2a,b to be about 35%, corresponding to ~−2% of MFIS.

Figure 2c depicts the normalized magnetization curves of composite measured along and perpendicular to the particles chains directions. The curves in Fig. 2c reveal a magnetic anisotropy of composite, which means that the easy and hard magnetization directions of composite coincide with the directions parallel and perpendicular to the particle chains, respectively, evidencing a preferential crystallographic orientation of the tetragonal lattice in the particles with the short *c*-axis along the chains direction. The crystallographic and spatial orientation of the particles inside of a silicone polymer with a stiffness value below 1 MPa (comparable to the twinning stress of particles) enabled the large macroscopic MFIS and MFIS exceptional features of the composite, as described below.

### Magnetostrain of the Ni-Mn-Ga/silicone composite

**Figure 3:**
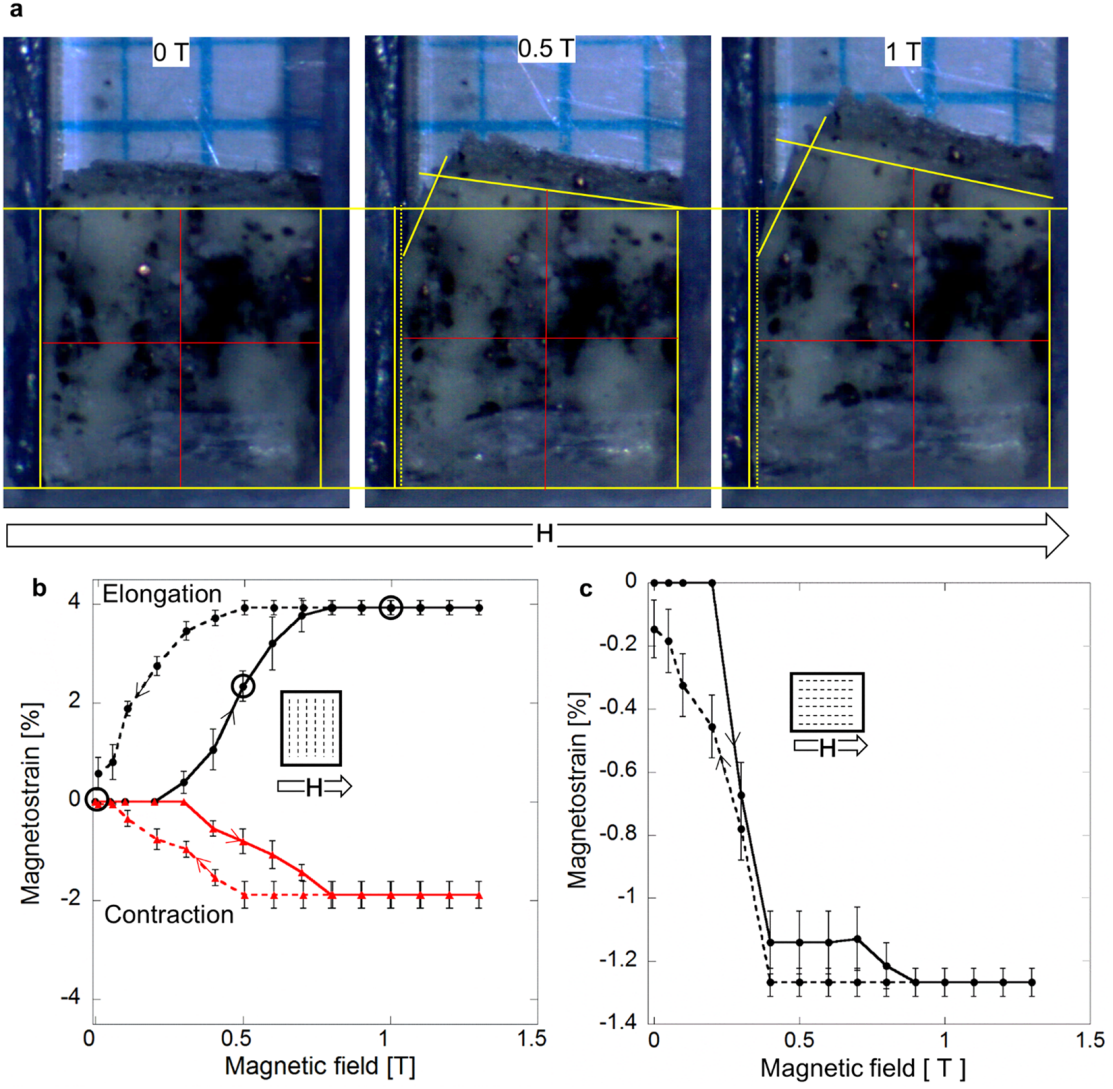
Magnetic field-induced deformation of Ni-Mn-Ga particles/silicone composite optically measured using ImageJ program. (**a**) Typical images of the composite sample (contoured by yellow color) inside of the holder with a reference grid and drawing method for calculation of its dimensions changes by the length evolutions of the red lines. (**b**,**c**) Magnetostrain curves showing the sample contraction along the magnetic field accompanied by its elongation in the orthogonal direction (solid curves, the open circles correspond to the three deformation stages in (**a**). Dashed curves depict the strain recovery as the magnetic field removes. Two different configurations: “magnetic field direction versus particles chain orientation” are schematically shown in (**b**,**c**). The composite deforms when the magnetic field reaches the value necessary to move twin boundaries in the particles (see Fig. 2). The strain recovery of the sample during field removal proceeds thanks to the reverse local stresses acting on the particles by a surrounding matrix.

The field-induced macroscopic deformation of a rectangular sample, cut from the 30 vol% Ni-Mn-Ga/silicone composite, was measured optically with a CCD camera. The sample was fixed by one face to a sample holder with the particles chains perpendicular to this face (Fig. 3a). The magnetic field from the electromagnet was manually increased/decreased in the range of 0–1.3 T in a step-wise manner (see Methods and Supplementary video).

In the first series of magnetostrain measurements (Fig. 3a,b), a field, H, was applied perpendicular to the particles chains as schematically shown in Fig. 3a. When the magnetic field reached a switching value, the embedded particles started to contract by rearranging their twin structure to align their short *c*-axis parallel to the field direction, whereas exhibiting a concurrent elongation in the orthogonal direction along the particles chains. The twin boundary movements terminated when the magnetization vector of particle was aligned along the magnetic field. The cooperative magnetic field-induced deformations of the individual particles resulted in the deformation of the entire sample, the effect which is found for the first time. As can be seen from Fig. 3a, the sample elongation is also accompanied by its attempt to bend towards one pole of the magnet, due to the contribution of magnetostatic force trying to rotate the sample as a whole.

Using images, such as shown in Fig. 3a, and selected geometrical procedure, the strains of composite sample in two orthogonal directions were calculated for each magnetic field. The plotted dependencies are depicted in Fig. 3b. The dependencies reveal that the variant reorientation in the particles starts at the switching field of about 0.3 T and proceeds until the magnetic moments are aligned along the field direction at about 0.7 T. Generally, the first-of-this-kind result deduced from the curves in Fig. 3b is two-fold: (a) the polymer composite in the field of about 0.7 T, applied perpendicularly to the sample texture, exhibited a large shape change: 4.0% in the elongation and −2% in the contraction; and (b) this MFIS was fully recoverable, with some hysteresis, after removal of the magnetic field. Fifteen times cycling reproduced MFIS data within uncertainties indicated in Fig. 3b by the error bars. The deformation of the same composite sample along the particle chains direction was also tested under the magnetic field applied in the same direction. Figure 3c demonstrates that the field-induced compressive deformation starts at about 0.2 T and proceeds up to its maximum value of −1.3% at 0.9 T, in a two-step manner while the strain recovery occurs in one step. Similar reduced values of MFIS and characteristic fields can be also observed in the bulk Ni-Mn-Ga single crystalline samples when they are partially constraint^2,24^.

Furthermore, according to Fig. 3b, the composite exhibited a 4% of MFIS, which can be compared to a maximum achievable strain in the Ni-Mn-Ga bulk single crystal (6%) at the same orientation between applied field and appropriate crystallographic direction^2^. Also the value of the switching field necessary to activate twin boundary motion, and the field value needed for their immobilization, equal to 0.3 T and 0.7 T, respectively, are similar in both cases^24^.

All the results in Fig. 3 demonstrate a unique phenomenon: an almost complete recovery of strain after removal of the magnetic field. This indicates a principal difference in the MFIS behavior between bulk single crystal (or foam) and composite because in the bulk samples this recovery is only possible when the external orthogonal force is applied. The origin of restoring force in the case of composite will be discussed below.

### Magnetostrain of individual particles in composite

**Figure 4:**
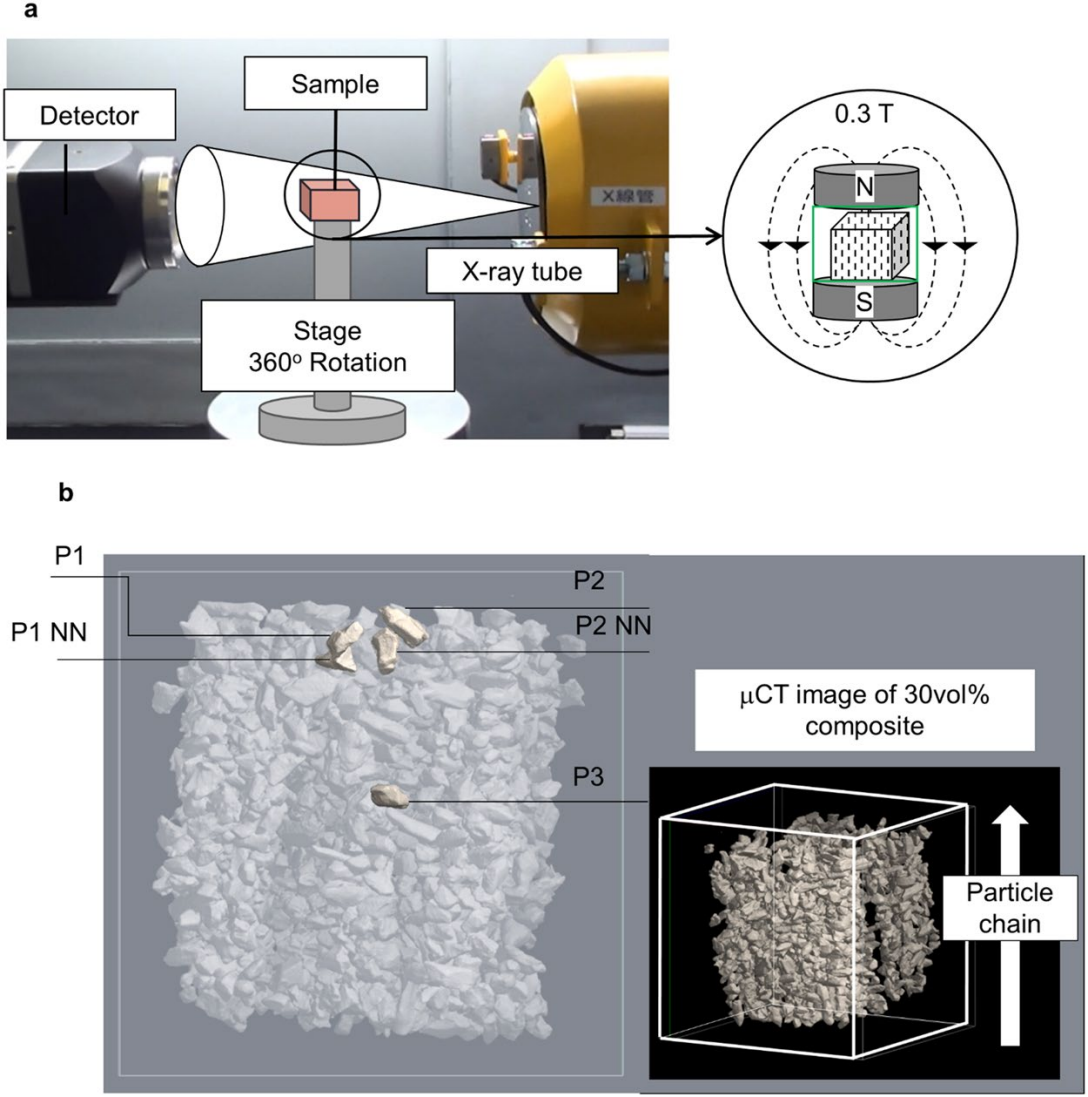
(**a**) Set up for the *in-situ* X-ray micro-computed tomography (μCT) observations under constant magnetic field of 0.3 T applied to the composite sample along the direction of particles chains; (**b**) 3D image of sample (right) and the location of selected particles for strain calculations (left). P1NN and P2NN are the nearest neighbors of the particles P1 and P2, respectively. P3 is isolated particle.

All aforementioned results about the magnetostrain of composite on the macroscale revealed clearly that the underlying mechanism of large MFIS is originated from the magnetic field-induced twin boundary motion in the particles. In order to ensure that embedded particles deform individually under the magnetic field, we have used the *in-situ* observations by X-ray micro-computed tomography (μCT) set up (Fig. 4a). Due to instrumental limitations, it was only possible to study the sample under a constant field of 0.3 T applied along the direction of particles chains.

Figure 4b provides 3D μCT images of sample indicating the location of selected particles for the strain calculations: two pairs with the close-distance layout and one relatively isolated particle. A particle strain was determined by the analysis of three-dimensional images obtained before application of the magnetic field^21,25^, under magnetic field and after its removal (see Supplementary Figs S1 and S2). Table 1 shows a summary of the strain component values along the chains direction exhibited by the individual particles, shown in Fig. 4b, under the magnetic field of 0.3 T and after its removal, together with the cross-section size and inter-particle distance. The results in Table 1 confirm an occurrence of the MFIS effect and its reversibility on the microscopic scale: the calculations, based on X-ray μCT images, revealed a ~−1.5% of compressive strain under 0.3 T and its disappearance after field removal in the particles P1 and P2. Actually, the similar values of strain have been observed in the present work for both the individual particle (~−2% estimated from Fig. 2a,b) and for the whole composite (~−1.3% from Fig. 3c). The isolated “free” particle P3 was deformed without recovery after removal of the magnetic field. Table 1 also demonstrates that particles P2 NN and P1 NN did not show appreciable strain under magnetic field. The reason could be their ideal crystallographic orientation, which means that their short *c*-axis is strictly aligned along the particle chain direction, as a result of fabrication. The other reason, probably more feasible, could be related to the appearance of the local stresses in the matrix leading to an increase of the effective stiffness of the latter (due to the inhomogeneous straining of the close-neighbor particles)^21^. These stresses, depending on the value, either block the particle deformation (case of P1 NN and P2 NN), or serve as a driving force for the deformation recovery (case of P1 and P2). Obviously, no such stresses were expected in the vicinity of isolated “free” P3 particle. Table 1 confirms a no-strain-recovery in this case.

**Table 1:** Magnetic field-induced strain component of the individual particles, shown in Fig. 4b, calculated along the direction of particles chains from the *in-situ* X-rays μCT images.

Particles	Cross- section size [μm]	Inter-particle distance [μm]	Strain under 0.3 T [%]	Strain after removal of field [%]
P1	209 ± 0.6	297 ± 1	−1.4 ± 0.3	0 ± 0.3
P1 NN	253 ± 0.6		−0.6 ± 0.3	+0.6 ± 0.3
P2	193 ± 0.6	280 ± 1	−1.5 ± 0.3	0 ± 0.3
P2 NN	238 ± 0.6		0 ± 0.3	0 ± 0.3
P3	190 ± 0.6	580 ± 1	−0.8 ± 0.3	−1.0 ± 0.3

Magnetic field of 0.3 T was applied and removed along the same direction.

## Discussion

The recoverable and repeatable large MFIS is an important finding in the present work. In the case of the bulk single crystal, similar behavior can be obtained only if a bias external force is applied^2,12^. In both cases, the hysteretic behavior of MFIS is attributed to the mechanical energy absorption during back and forth movements of the twin boundaries.

The restoring force in the case of composite is assumed to be produced by the internal local stresses arising in the matrix surrounding particles, as a result of their field-induced deformations and strengthening of the interactions between them. This hypothesis is justified by the results of the stress-strain behavior of Ni-Mn-Ga/silicone composite on a microscale studied in ref.^21^. It was shown that the particles interactions produced local changes of the elastic stiffness of the surrounding matrix, whereby the drastic changes of slopes on the dependencies “particle strain versus applied compressive strain” were observed^21^.

It is worth noting that the nonmagnetic Au–Cd and Cu-based shape memory alloys exhibit the reversible twin boundaries rearrangements in the martensitic phase under application and removal of the mechanical stress^26–28^. This effect is commonly known as the rubber-like behavior of martensite. In the present work we found a magnetic analog of this phenomenon which can be now termed as a magnetic field-induced rubber-like behavior of the FSMA/polymer composite. Such a magnetostrain-active composite can be also called as a magnetoshape resin featuring different functionality than the magnetic elastomers^29^.

In summary, a magnetic field-induced rubber like behavior has been found for the first time in Ni-Mn-Ga/silicone composite, which could repeatedly deform by about 4.0% of elongation and −2.0% of contraction along and perpendicular to the particle chains, respectively, under magnetic field of about 0.7 T applied along the perpendicular-to-chain direction. Moreover, a reversible strain, about −1.5%, of the individual particles under magnetic field of 0.3 T was determined by the *in-situ* X-ray μCT imaging analysis. For comparison, the macroscopic magnetostrain in our material is 20 times larger than in the commercial magnetostrictive material Terfenol-D^30^. The remarkably high magnetostrain response of composite was achieved owing to the large magnetic field-induced shape changes of the single-grain particles in silicone polymer with a sufficiently low stiffness (<1 MPa). The selected polymer presented a soft enough solid state environment ensuring a magnetoshaping of particles due to the twin boundaries motion. The strikingly unusual effect of the fully reversible deformation of both the individual particles and composite as a whole, was explained in terms of the local stresses in polymer accumulated near close-neighboring particles during field application and local stresses relaxation during field removal. The utilization of the elaborated composite material exhibiting a magnetic field-induced rubber-like effect can significantly simplify the actuation or sensing devices since no external biasing force is required to reset it after removal of the magnetic field. The magnetostrain-active composite and its unusual properties suggest novel solutions for the development of the low cost magnetic actuators and sensors for haptic applications.

## Methods

### Fabrication of Ni-Mn-Ga magnetostrain-active particles and composite

A polycrystalline ingot with the composition of Ni_50_Mn_28_Ga_22_+0.03at%Bi was prepared by the arc melting under argon atmosphere. The ingot was homogenized at 1000 °C for 24 hours followed by a water quenching. Particles were fabricated by the careful disintegration of ingot at 300 °C (above melting point of bismuth, 271 °C) into the individual grains. As bismuth segregated at the grain boundaries, the decreasing of the adhesion force between grain boundaries, due to a liquid state of bismuth, resulted in the intergranular easy-fracture, whereby the single crystalline grains with the clean and shiny faces were obtained after mechanical cleaving^17,20^. The powder was divided by the sieves into three fractions having different size of particles: below 75 μm, 100 μm–250 μm and over 250 μm, which were used for the measurement of the transformation temperatures, fabrication of composite and optical observation of the twin rearrangements under magnetic field, respectively. To eliminate any residual stress and improve atomic order, powder was encapsulated under argon atmosphere in the quartz ampoule and held at 800 °C for 1 hour, followed by the furnace cooling down to the room temperature. The martensitic transformation and Curie temperatures were determined by Differential Scanning Calorimetry (DSC-60 Shimadzu instrument) during heating and cooling ramps (5 °C/min) in the temperature range of 0–150 °C. The structural characterization of powder at room temperature was performed by the X-ray diffractometer PANalytical X´pert Pro Galaxy, Philips, using CuK_α_ radiation. Analysis of the X-ray diffraction pattern was carried out with a FullProf program.

The large particle for the optical observation of a martensitic variants reorientation under magnetic field was mechanically polished using #4000 sand paper at 80 °C in austenite state, then, cooled down to assure martensitic state at the room temperature. The permanent magnets were configured to produce a field of 0.5 T during observation.

To fabricate composite, first, the commercial silicone rubber ELASTOSIL M4400 was mixed, by the ratio of 100:3, with a curing agent T40 Wacker Catalyst from Wacker A.G. Silicone. According to manufacturer, the hardness of this silicone rubber after curing should be approximately equal to 23 in Shore A scale. Its elastic stiffness, of about 0.5 MPa, was determined as a slope of the “stress-strain” dependence in Supplementary Fig. S3 measured by a Shimadzu Autograph AG-500N testing machine. Second, the Ni-Mn-Ga powder with a 30 vol.% fraction was mixed with a slurry silicone inside of the acrylic mold. The curing of composite was carried out for 8 hours under a field of 1 T to align particles in the chains and create a cooperative crystallographic texture, where the easy-magnetization short *c*-axis in particles was oriented along the particles chains direction. For the magnetomechanical measurements, the samples measuring of 3.5 × 4 × 4 mm^3^ were cut from composite.

### Measurements procedure

The three-dimensional imaging of the embedded particles in silicone matrix was performed by a Comscan Scan Xmate X-ray μCT set-up. The sample was scanned under 120 kV and 95 μA with 2 × 2 binning mode and 900 projections number. Duration of the one full scan was 8 hours. CCD camera with 4000 × 2672 pixels enabled the image resolution of 1.67 μm. To confirm the cooperative alignment of the easy magnetization *c*-axis in particles, resulting in the magnetic anisotropy of the composite sample, the “magnetization versus field” measurements, in which the magnetic field applied along and perpendicular to the particle chains directions, respectively, were performed at room temperature using a Vibrating-Sample Magnetometer (VSM, TM-VSM1530-HGC-D, Tamakawa Co., Ltd).

The acrylic sample holder was designed to investigate the macroscopic deformation of composite under a magnetic field. A panel with the precise 1 × 1 mm^2^ grid size was placed behind the sample for the scale calibration of optical images. The sample holder was mounted between the poles of the electromagnet with a help of wooden arm. A 400 mm, long-range focus optical CCD camera (LRA130XGA-3E, Shodensha) was adjusted to avoid an influence of magnetic field on the parameters of camera and get the image resolution of 1280 × 1024 pixels. In the first series of observation of macroscopic deformation, the magnetic field was applied perpendicular to the particle chains direction. The magnetic field strength was manually changed in the range of 0–1.3 T. The holding time before changing to the next field step was 3 minutes followed by taking snap-shot of sample. The magnetomechanical cycling was made fifteen times.

A second series of observations was carried out by applying field along the particle chains direction. Then, in the same “field – sample” orientation, the sample was studied by an *in-situ* X-ray μCT imaging. As illustrated in Fig. 4a, the special sample holder comprised also two Neodymium permanent magnets producing field of 0.3 T.

To determine magnetostrain value, each photographic image of composite sample, taken in a single-field-step, was implemented in the ImageJ program. Since the units measured by this program have been pixels, all images were calibrated to the metric scale with a reference to a grid in the sample holder. The resolution after calibration was 100–120 pixels/mm. The magnetostrain was calculated as follows:

1 $$\varepsilon =(\frac{{\ell }_{i}-{\ell }_{0}}{{\ell }_{0}})\times 100 \% ,$$

where ${\ell }_{0}$ and ${\ell }_{i}$ is the length of red line shown in Fig. 3a before and after deformation, respectively. The red line is drawn in the middle of the schematic frame of sample indicated by yellow.

### Analysis of the field-induced deformation of individual particles in composite using X-ray μCT 3D images tracking method

For such an analysis we have used the same calculation method as described in ref.^25^. Here we shortly present the fundamental concept of this method which is summarized in Supplementary Fig. S1. According to continuum mechanics, the deformation of material can be obtained by the tracking of the position of specific point before and after deformation. The positions of specific points in the X-ray μCT 3D images of particle(s) taken before and after deformation are attributed to the field- or stress-induced distance changes between the mentioned points. At least six specific points should be selected to achieve a sufficient resolution and high accuracy of the calculations (see Supplementary Fig. S2).

Let **r**_**j**_ and **R**_**j**_ be the coordinate vectors in Cartesian coordinates of each specific point *j* measured before and after deformation, respectively:

2 $${{\bf{r}}}_{{\bf{j}}}=\{({x}_{j},{y}_{j},{z}_{j})\}|1\le j\le N$$

3 $${{\bf{R}}}_{{\bf{j}}}=\{({X}_{j},{Y}_{j},{Z}_{j})\}|1\le j\le N,$$

where *N* is the total number of selected points on the particle surface in the 3D images.

According to ref.^31^, the coordinates of the specific points after deformation stage, ${{\bf{R}}{\boldsymbol{^{\prime} }}}_{{\bf{j}}}$, can be calculated by multiplying **r**_**j**_ and deformation gradient, **F**:

4 $${{\bf{R}}{\boldsymbol{^{\prime} }}}_{{\bf{j}}}={\bf{F}}{{\bf{r}}}_{{\bf{j}}},$$

where **F** is the deformation gradient that, in turn, can be expressed in arbitrary variables as:

5 $${\bf{F}}=[\begin{array}{ccc}a & b & c\\ d & e & f\\ g & h & i\end{array}].$$

To minimize the error, the deviation parameter, **d**, obtained as a result of the difference between the measured, **R**_**j**_, and the calculated, ${{\bf{R}}{\boldsymbol{^{\prime} }}}_{{\bf{j}}}$, values in the reference coordinate system, was refined by the least square method using formula:

6 $${{\bf{d}}}^{2}={\sum _{j=1}^{N}({\bf{F}}{{\bf{r}}}_{{\bf{j}}}-{{\bf{R}}}_{{\bf{j}}})}^{2}.$$

A reliable determination of **F** can be achieved if minimized value of **d**^2^ is set to zero or close to it. The martensitic variants reorientation in Ni-Mn-Ga can be considered as a plastic deformation, so the volume conservation principle should be valid. In other words, the determinant of deformation gradient, det**F**, should be equal to 1 with a correction factor, *k*, related to the difference in the calculated volume before and after deformation, which is expressed as:

7 $$k={(\det {\bf{F}})}^{-\frac{1}{3}}.$$

A polar decomposition was used to decompose the rotation matrix, **Q**, and stretch tensor, **V**, from the deformation gradient. Finally, the particle strain, ε, was obtained from the equation:

8 $${\boldsymbol{\varepsilon }}={\bf{V}}-{\bf{I}},$$

where **I** is the identity matrix. The component of **ε**, which is parallel to the magnetic field direction, was considered in this study.

## Supplementary information

Particles distribution in 30vol% Ni-Mn-Ga particles/silicone composite taken by X-ray μCT

Measurement of magnetostrain of 30vol% Ni-Mn-Ga particles/silicone composite

Supplementary information

## Data Availability

Authors confirm that all relevant data were included in the paper and/or in the supplementary information files.
