# Supplementary material for: Magnetic field-induced rubber-like behavior in Ni-Mn-Ga particles/polymer composite
Source: Sci Rep. 2019 Mar 5;9:3443. doi: 10.1038/s41598-019-40189-2 (PMC6401090; doi:10.1038/s41598-019-40189-2)
Supplement: Supplementary file 3 — Supplementary information [file 41598_2019_40189_MOESM3_ESM.pdf]

Magnetic field-induced rubber-like behavior in Ni-Mn-Ga particles/polymer composite

P. Sratong-on, V.A. Chernenko, J. Feuchtwanger and H. Hosoda

Supplementary Figures

Supplementary Figure S1:
Schematic diagram describing image tracking procedure for the calculation of the particle strain.

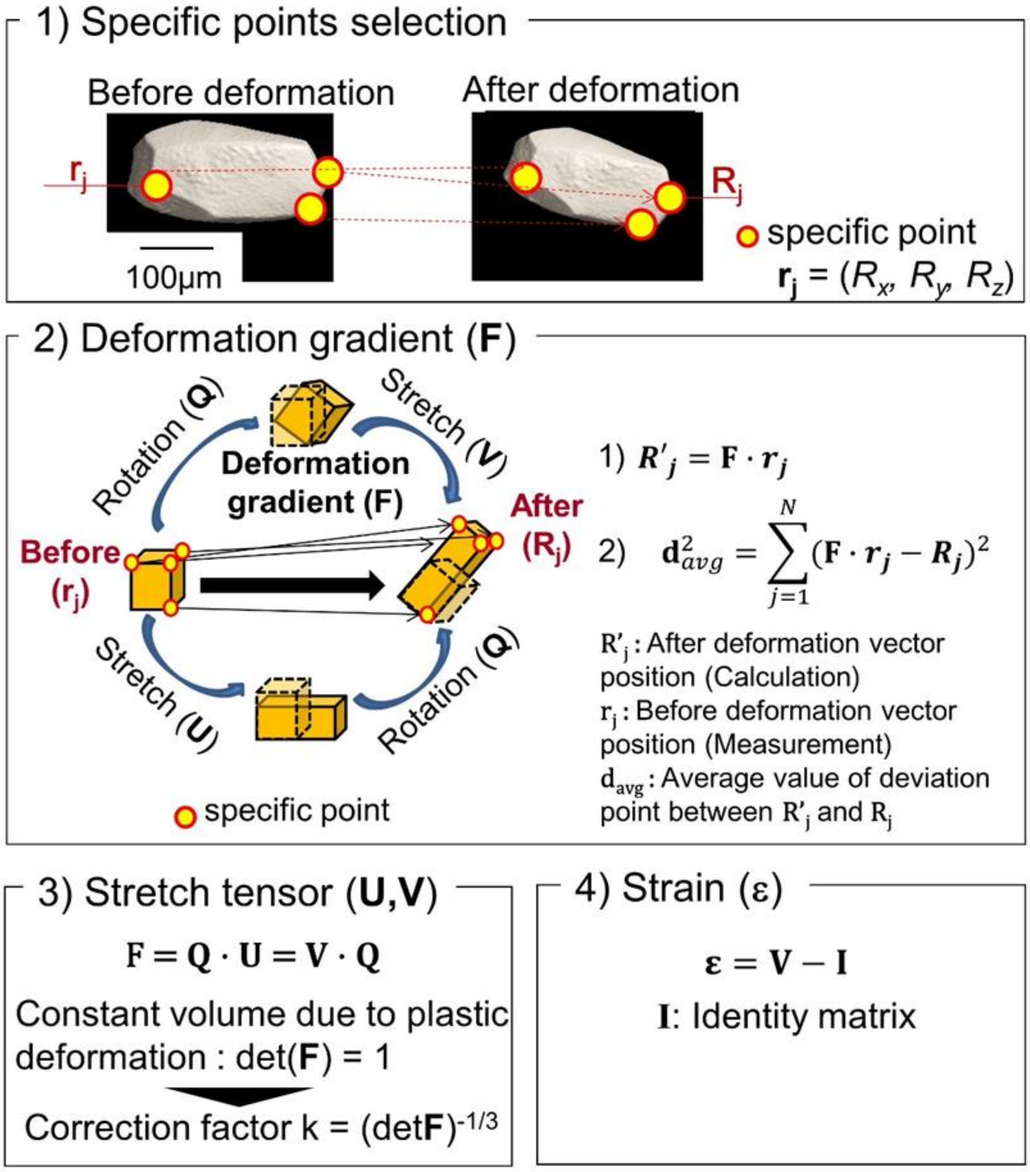

Supplementary Figure S2:

An accuracy of the particle strain calculations by image tracking method.

(a) Frequency of calculations as a function of strain along loading axis in case of six specific points

(b) The voxel of μCT image. L is the image resolution of μCT

(c) The standard deviation (SD) of particle strain determined by image tracking as function of the number of selected specific points and the μCT image resolution

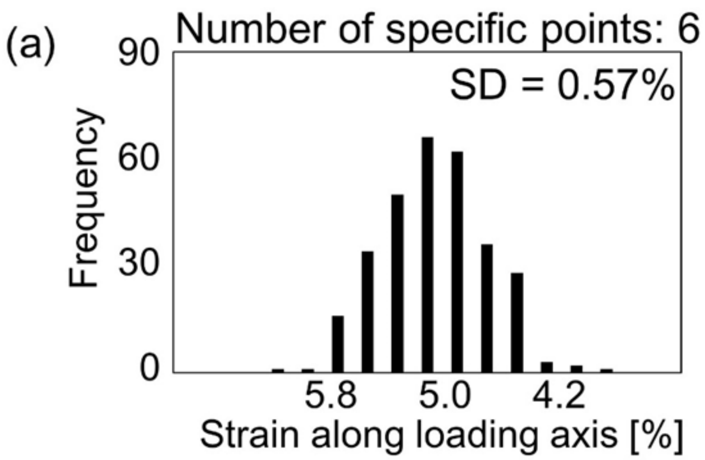

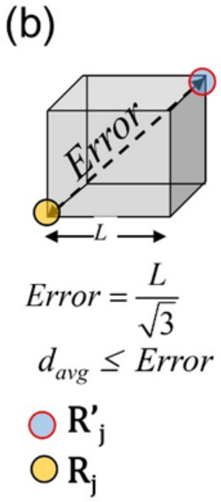

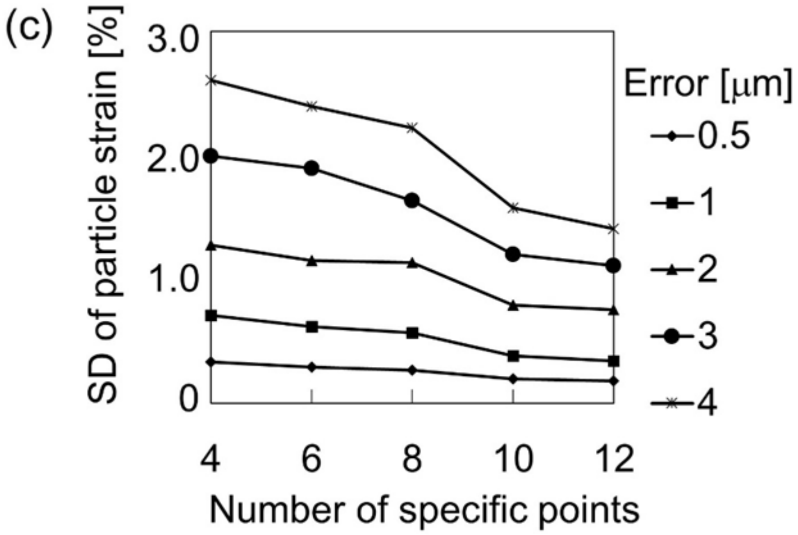

Supplementary Figure S3:

Stress-strain dependence of the prepared silicon rubber ELASTOSIL M 4400 with Shore A = 23.

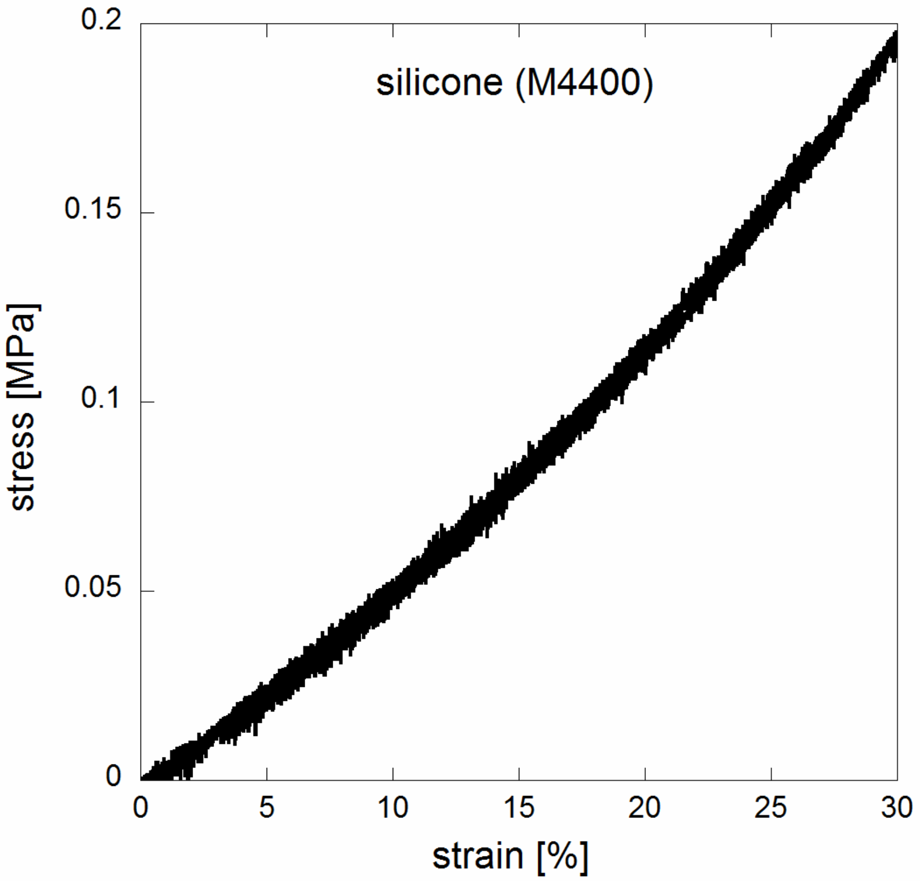

Supplementary Videos

1) Particles distribution in 30vol% Ni-Mn-Ga particles/silicone composite taken by X-ray μCT.
2) Measurement of magnetostrain of 30vol% Ni-Mn-Ga particles/silicone composite
